# Characterization of Syphilitic Chorioretinitis as a White Dot Syndrome with Multimodal Imaging: Case Series

**DOI:** 10.3390/diagnostics15030369

**Published:** 2025-02-04

**Authors:** Robert J. Contento, Neha Gupta, Mark P. Breazzano

**Affiliations:** 1 Norton College of Medicine, SUNY Upstate Medical University, Syracuse, NY 13210, USA; contentr@upstate.edu; 2 Department of Ophthalmology & Visual Sciences, SUNY Upstate Medical University, Syracuse, NY 13210, USA; neha03010@gmail.com; 3 Department of Ophthalmology, New York Eye & Ear Infirmary, Icahn School of Medicine at Mount Sinai, New York, NY 10003, USA; 4 Flaum Eye Institute, Department of Ophthalmology, University of Rochester Medical Center, Rochester, NY 14642, USA

**Keywords:** syphilis, syphilitic chorioretinitis, ultra-widefield imaging, fundus autofluorescence, white dot syndrome, multimodal imaging

## Abstract

**Background/Objectives**: To investigate the role of multimodal imaging, including ultra-widefield fundus autofluorescence (UWFAF), in diagnosing and monitoring syphilitic chorioretinitis, focusing on the detection of placoid appearance and white dots/spots. We aim to classify syphilitic chorioretinitis as a white dot syndrome, given evident features in the context of recent case reports and previously unavailable multimodal imaging. **Methods**: This single-institution study was conducted as a consecutive, observational case series. Five eyes from three patients were diagnosed with syphilitic chorioretinitis using multimodal imaging, including ultra-widefield pseudocolor fundus photography and intravenous fluorescein angiography, UWFAF, and swept-source optical coherence tomography, upon laboratory results. **Results**: In all five eyes with serologically confirmed syphilitic chorioretinitis, UWFAF revealed hyperautofluorescent white dots and spots scattered in the fundus, a finding minimally apparent with fluorescein angiography. Two eyes did not show evidence of classic placoid lesions. The hyperautofluorescence resolved after standard neurosyphilis treatment with intravenous course of penicillin. **Conclusions**: The presence of dots and spots identified through UWFAF may indicate syphilitic chorioretinitis and support its classification as a white dot syndrome. Based on the presence of hyperautofluorescent placoid lesions in some but not all cases with dots and spots, this study highlights the utility of multimodal imaging, including the more recent availability of UWFAF, in diagnosing syphilitic chorioretinitis. Future research is needed to determine whether the dots and spots in syphilitic chorioretinitis represent direct spirochete infiltration or a secondary inflammatory response.

## 1. Introduction

Syphilis is a sexually transmitted infection caused by the spirochete *Treponema pallidum*, known for its ability to affect multiple body systems and masquerade as many diseases [1]. Ocular involvement can occur in the anterior and posterior segments of the eye at any stage of the infection, whether it be primary, secondary, or tertiary syphilis. This disease progression makes early recognition and treatment critical [2]. Syphilitic chorioretinitis, the most common posterior segment presentation of syphilis, is a challenging diagnosis due to its diverse presentations and has been increasingly recognized among the resurgence of syphilis cases in the United States. An elevenfold rise in congenital syphilis diagnoses and a marked increase in syphilitic uveitis-related hospitalizations in the last decade show the growing public health burden [3,4,5]. Rapid and accurate diagnosis is essential to prevent severe visual complications and systemic disease progression [6,7,8].

When presenting as posterior uveitis, syphilitic chorioretinitis usually involves damage to the retinal pigment epithelium (RPE) and neurosensory retina. Syphilitic outer retinitis (SOR) is a type of syphilitic chorioretinitis that can present as punctate inner retinitis, outer retinitis, or placoid lesions. These presentations can range from retinal hemorrhages and retinal vessel inflammation to necrotizing retinitis and conditions mimicking white dot syndromes [9,10,11,12,13,14,15]. This variability in presentation shows the diagnostic challenges clinicians face and the need for advanced imaging techniques to characterize retinal and choroidal involvement. Ultra-widefield fundus autofluorescence (UWFAF) has become a noninvasive imaging modality capable of capturing peripheral retinal findings and localizing inflammation and structural damage, providing diagnostic insight into syphilitic chorioretinitis [16,17].

Widefield and ultra-widefield imaging have revolutionized retinal imaging over the past century. The first fundus camera was introduced by Carl Zeiss and J.W. Nordensen in 1926 and evolved to provide the standard retinal view of 30 degrees [18,19]. The introduction of non-contact ultra-widefield systems, such as Optos California (Optos Inc., Marlborough, MA, USA) scanning laser ophthalmoscopy in the early 2000s, enabled two-hundred-degree imaging, covering over 80 percent of the retina with minimal preparation [2]. Alternative modern systems like the Zeiss Clarus 500 (Carl Zeiss Meditec USA Inc., Dublin, CA, USA) introduced true-color imaging while maintaining some peripheral capture [20,21]. Regardless of the specific platform, ultra-widefield retinal imaging is essential in diagnosing and managing retinal and associated systemic diseases.

UWFAF has proven useful in identifying distinct features of syphilitic chorioretinitis, including hyperautofluorescent regions that correspond to RPE disruption. This imaging modality facilitates the visualization of both classic placoid lesions and scattered hyperautofluorescent spots, which likely represent disrupted luteal pigment in the neurosensory retina [22,23]. These hyperautofluorescent dots and spots, resembling the features of white dot syndromes, may serve as a diagnostic marker for syphilitic chorioretinitis. Additionally, UWFAF enables clinicians to monitor disease resolution post-treatment by visualizing structural and functional recovery [24,25].

White dot syndromes are a group of inflammatory chorioretinopathies defined by the presence of multiple discrete white lesions located within the retinal layers and choroid [26]. Common symptoms include photopsia, floaters, blurred vision, decreased night vision, and visual field loss, with the syndromes most often affecting young and healthy adults [27]. Despite their shared clinical features, white dot syndromes are diverse and include conditions such as Multiple Evanescent White Dot Syndrome (MEWDS), Acute Retinal Pigment Epitheliitis (ARPE), Acute Posterior Multifocal Placoid Pigment Epitheliopathy (APMPPE), Multifocal Choroiditis and Panuveitis (MCP), Acute Zonal Occult Outer Retinopathy (AZOOR), Birdshot Chorioretinopathy, Serpiginous Choroidopathy, and Punctate Inner Choroidopathy (PIC) [12,26]. While the etiology of white dot syndromes remains largely unknown, many cases have been linked to a preceding viral prodrome, suggesting an infectious or autoimmune trigger [28].

Given the similarities in clinical and imaging findings, particularly with UWFAF, this study seeks to characterize syphilitic chorioretinitis as a white dot syndrome. By characterizing the unique imaging features of syphilitic chorioretinitis, this study compares its diagnostic overlap with white dot syndromes and emphasizes the value of UWFAF in detecting and monitoring the condition. UWFAF is used in a multimodal approach alongside swept-source optical coherence tomography (OCT), ultra-widefield intravenous fluorescein angiography (IVFA), and ultra-widefield pseudocolor fundus photography (CFP) for detailing associated clinical features. Ultimately, this investigation aims to support the integration of UWFAF into a conventional diagnostic paradigm for syphilitic chorioretinitis and advocate for its recognition as a member of the white dot syndrome spectrum.

## 2. Materials and Methods

This consecutive, observational case series was designed in accordance with CARE Guidelines by EQUATOR Network for three patients diagnosed with syphilitic chorioretinitis. Patients were evaluated in a retina subspecialty clinic at a single institution. Patient consent was waived due to minimal patient risk and study design. The study was exempt from review by the governing Institutional Review Board at Crouse Hospital (Syracuse, NY, USA). All procedures were reviewed and in accordance with tenets of the Declaration of Helsinki.

**Figure 1:**
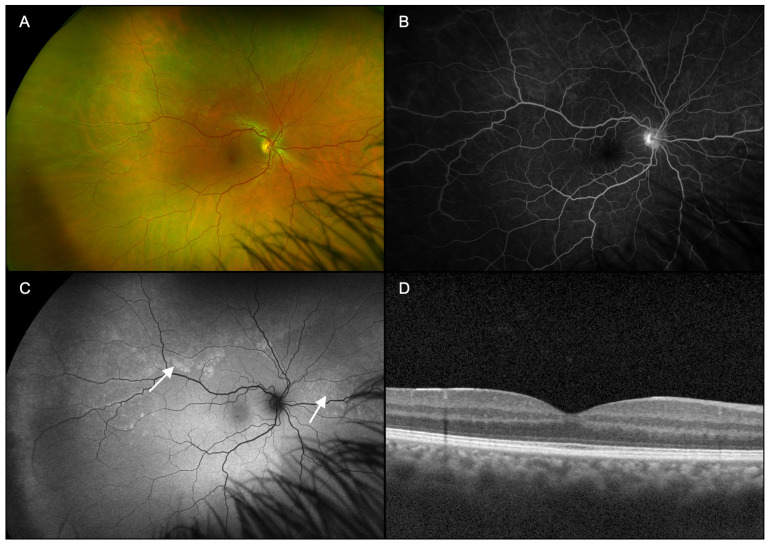
Case 1, right eye (OD). (**A**): Ultra-widefield pseudocolor fundus photography demonstrates numerous, subtle peripheral white dots, some coalescing into larger spots, possibly into placoid lesions. (**B**): Lase-phase fluorescein angiography demonstrates optic nerve head leakage centrally. (**C**): Fundus autofluorescence colocalizes with greater clarity the scattered white dots seen on fundus examination as discrete regions of hyperautofluorescence (arrows). (**D**): Swept-source optical coherence tomography did not show evidence of retinal pigment epithelium or ellipsoid zone disruption in the macula.

**Figure 2:**
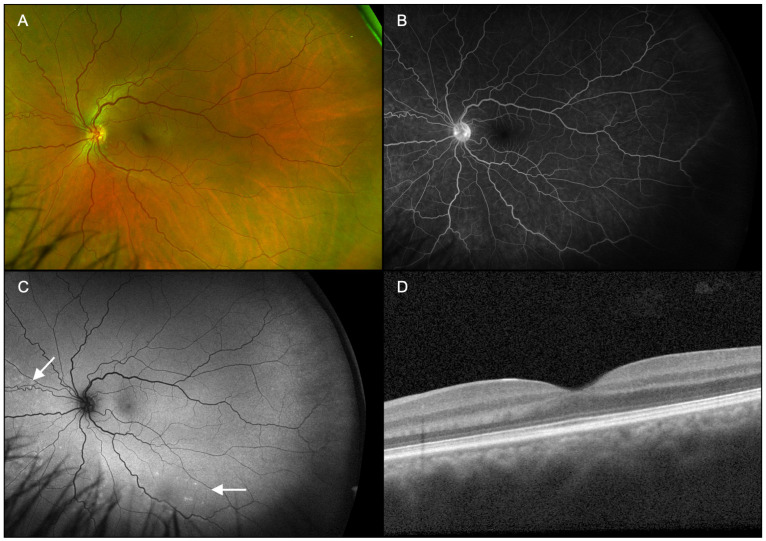
Case 1, left eye (OS). (**A**): Ultra-widefield fundus pseudocolor photography shows nearly invisible peripheral white spots, fewer in number than the fellow eye, yet without placoid lesions. (**B**): Late-phase fluorescein angiography demonstrates active optic nerve head leakage. (**C**): Fundus autofluorescence colocalizes with the white dots seen on fundus examination as discrete foci of hyperautofluorescence (arrows). (**D**): Swept-source optical coherence tomography of the macula did not show evidence of retinal pigment epithelium or ellipsoid zone disruption.

**Figure 3:**
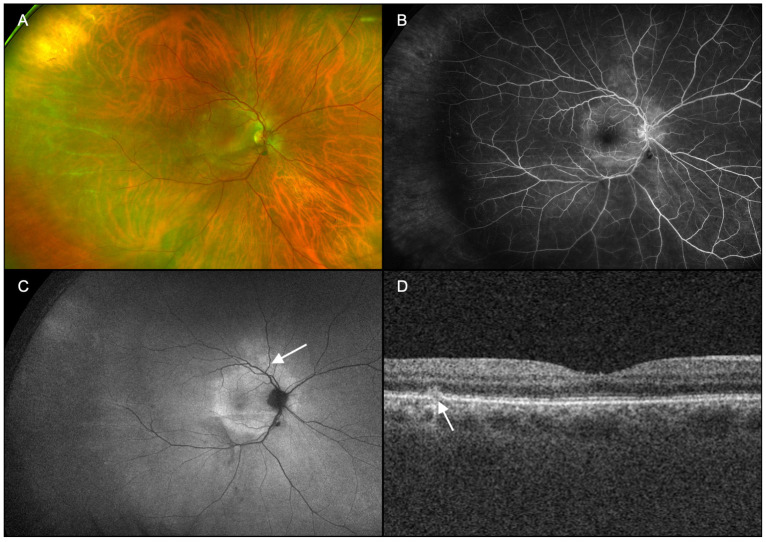
Case 2, right eye (OD). (**A**): Ultra-widefield pseudocolor fundus photography showed retinal pigment epithelium changes and sclerotic vessels temporally. (**B**): Fluorescein angiography demonstrated leakage at the optic nerve head and hyperfluorescence corresponding to the areas of retinal pigment epithelial changes seen on dilated exam. (**C**): Fundus autofluorescence revealed a large placoid lesion with neighboring coalesced and isolated dots and spots of hyperautofluorescence (arrow). (**D**): Swept-source optical coherence tomography of the macula showed evidence of disruption to the ellipsoid zone and retinal pigment epithelium/Bruch membrane with an outer retinal hyperreflective lesion (arrow).

**Figure 4:**
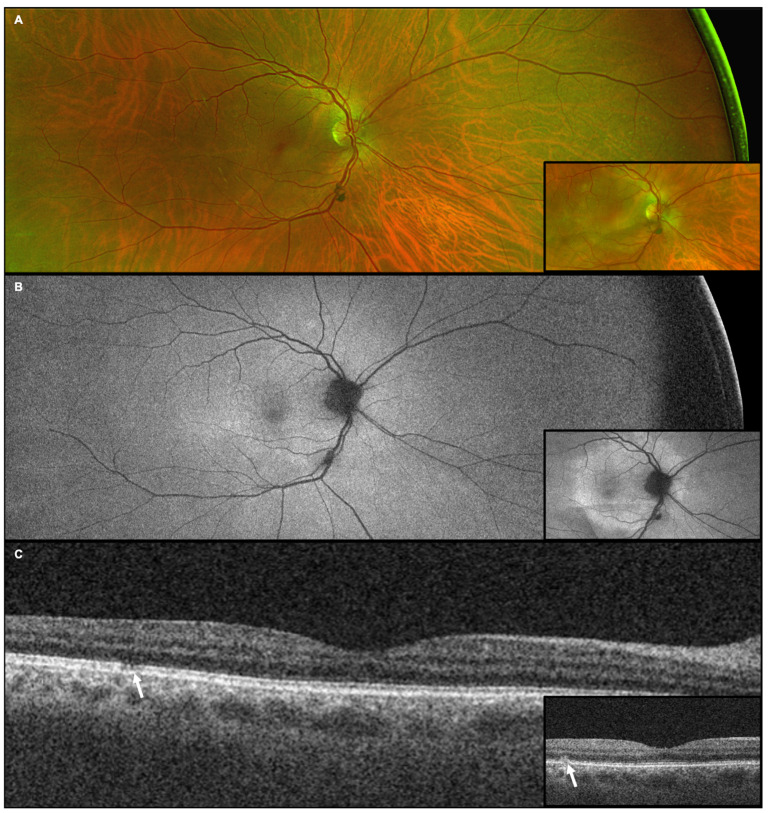
Resolution of outer retinal disruption and white dots and spots are seen after systemic treatment of syphilis in *Case 2*. (**A**): Ultra-widefield pseudocolor fundus photography reveals resolution of the coalesced white dots following penicillin administration. (**B**): Fundus autofluorescence demonstrates nearly complete resolution of hyperfluorescence. (**C**): Swept-source optical coherence tomography of the macula shows improvement in the outer retinal architecture (arrow), including adjacent areas of prior ellipsoid zone disruption. Insets compare respective imaging from initial presentation to show improvement following treatment.

**Figure 5:**
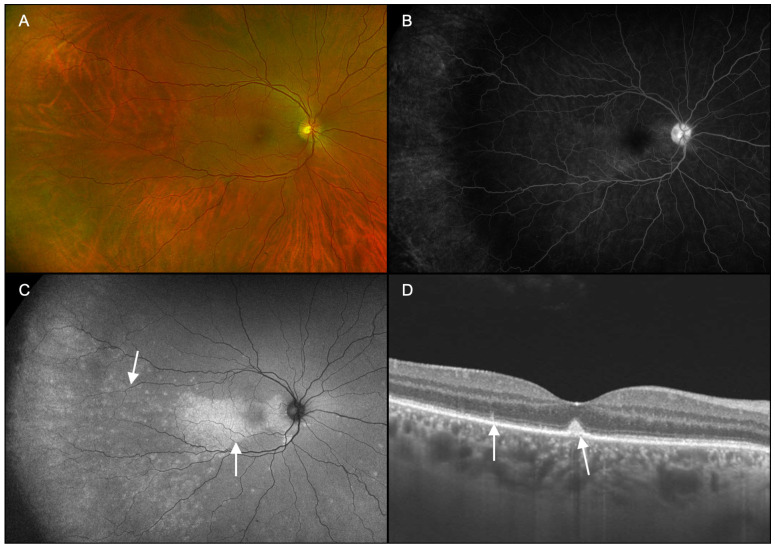
Case 3, right eye (OD). (**A**): Ultra-widefield pseudocolor fundus photography reveals vascular attenuation and retinal pigment epithelial changes in addition to subtle white dots and spots, some coalesced. (**B**): Fluorescein angiography demonstrates leakage consistent with active vasculitis. (**C**): Fundus autofluorescence shows numerous scattered dots of hyperautofluorescence extending into the periphery and adjacent to a macular placoid lesion (arrows). (**D**): Swept-source optical coherence tomography showed a conical, outer foveal hyperreflective lesion extending from retinal pigment epithelium/Bruch membrane and disrupting ellipsoid zone as well as a thickened choroid. Several other foci of ellipsoid zone disruption are also observed (arrows).

**Figure 6:**
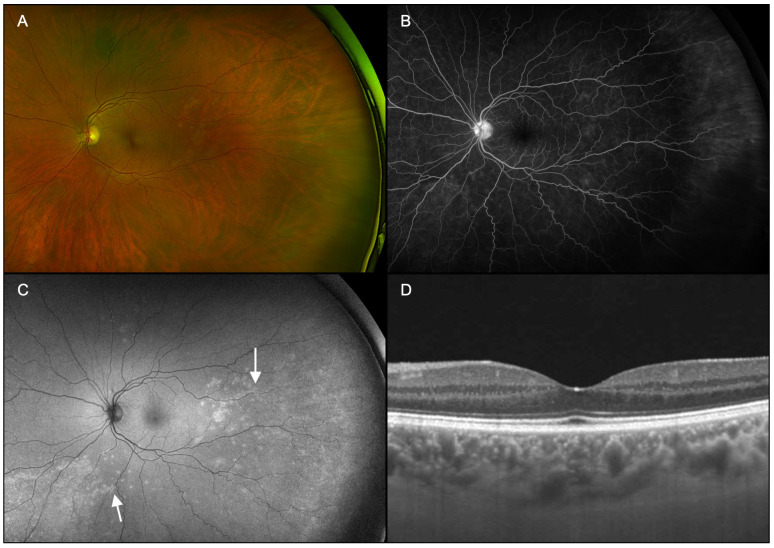
Case 3, left eye (OS). (**A**): Ultra-widefield pseudocolor fundus photography reveals subtle, small whitish lesions throughout the fundus with vascular attenuation and retinal pigment epithelial changes. (**B**): Fluorescein angiography demonstrated leakage of the optic nerve and vascular leakage consistent with active vasculitis. (**C**): Fundus autofluorescence demonstrated scattered dots of hyperautofluorescence including a placoid lesion in the periphery (arrows). (**D**): Swept-source optical coherence tomography showed a thickened choroid without any outer retinal disruption in the central macula.

Patients underwent ophthalmic examination including measurement of best corrected visual acuity (BCVA), dilation with topical tropicamide (1%) and phenylephrine (2.5%) followed by multimodal imaging (Figure 1, Figure 2, Figure 3, Figure 4, Figure 5 and Figure 6), including ultra-widefield CFP (Figure 1A, Figure 2A, Figure 3A, Figure 5A and Figure 6A), IVFA (Figure 1B, Figure 2B, Figure 3B, Figure 5B and Figure 6B), UWFAF (Figure 1C, Figure 2C, Figure 3C, Figure 5C and Figure 6C), and swept-source optical coherence OCT (Figure 1D, Figure 2D, Figure 3D, Figure 5D and Figure 6D). When available, testing was performed before (Figure 4 insets) and after neurosyphilis treatment with intravenous penicillin, including ultra-widefield CFP (Figure 4A), UWFAF (Figure 4B), and swept-source OCT (Figure 4C).

## 3. Results

### 3.1. Case 1

A 42-year-old man presented with a 2-month history of progressively worsening floaters in both eyes. The patient denied any significant past medical history. On presentation, BCVA was 20/30-2 in the right eye and 20/25 in the left eye. Anterior segment examination was normal in both eyes. On dilated examination, 1+ vitreous cell was noted in the right eye, and subtle white spots were found in the periphery, right greater than the left eye, without the presence of any placoid lesions (Figure 1A and Figure 2A). Imaging on presentation included swept-source OCT, ultra-widefield CFP, ultra-widefield IVFA, and UWFAF. FA showed active leakage from the optic nerve head in both eyes (Figure 1B and Figure 2B). UWFAF colocalized with the scattered white dots seen on fundus examination as discrete regions of hyperautofluorescence in the superotemporal region OD (Figure 1C) and inferonasally OS (Figure 2C). OCT did not demonstrate evidence of RPE or ellipsoid zone disruption in the macula; however, peripheral scans were not obtained in areas of involvement (Figure 1D and Figure 2D).

The patient underwent an infectious and inflammatory serological workup and was diagnosed with syphilis. The patient received treatment including intravenous penicillin G (24 million units/per day) for 14 days. Six months later, there was complete regression of the white dots in both eyes, and subsequent IVFA showed resolution of the bilateral disc leakage.

### 3.2. Case 2

A 67-year-old man with a past medical history of hypertension presented with a 1-month history of intermittent floaters in the right eye. BCVA was 20/20 in the right eye. Anterior segment examination was unremarkable. On dilated examination, 1+ vitreous cell and a hyperemic disc with sharp margins were noted in the right eye. The macula showed retinal pigment epithelial changes, ghost vessels, and perivascular sheathing temporally (Figure 3A).

IVFA demonstrated leakage at the optic nerve head and hyperfluorescence corresponding to the areas of retinal pigment epithelial changes seen on dilated exam (Figure 3B). UWFAF revealed a hyperautofluorescent placoid lesion with coalesced dots and spots of hyperautofluorescence in the macula that extended past the optic nerve head (Figure 3C). OCT macula showed evidence of hyperreflective opacities consistent with vitreous inflammation, as well as disruption and attenuation of the ellipsoid zone adjacent to the outer retinal hyperreflective lesion (arrowed site in Figure 3D). The extent of ellipsoid zone disruption, rather than the outer retinal hyperreflective lesion, likely corresponds to the more diffuse, hyperautofluorescent signal from disrupted luteal pigment in the macula consistent with its placoid appearance. Imaging studies in the left eye appeared normal. After extensive serological workup, the patient was diagnosed with syphilitic chorioretinitis in the right eye.

One month after treatment with intravenous penicillin G (24 million units/per day for 14 days), there was an improvement in symptoms and chorioretinal lesions (Figure 4). UWFAF appears to demonstrate resolution of both the coalesced and isolated white dots (Figure 4B) with greater clarity than ultra-widefield CFP (Figure 4A). OCT macula also revealed improvement to the ellipsoid zone architecture in the right eye (Figure 4C).

### 3.3. Case 3

A 56-year-old man with a past medical history of hypertension, hyperlipidemia, and thyroid disease presented with gradually worsening vision in his right eye associated with a central “cloudy spot” and floaters. BCVA was 20/50 in the right eye and 20/20 in the left eye.

A complete ophthalmic examination revealed trace cells in the vitreous, diffuse retinal vessel attenuation, and central and temporal RPE changes in both eyes (Figure 5A and Figure 6A). IVFA demonstrated late leakage of the optic nerve and vascular leakage consistent with active vasculitis in both eyes (Figure 5B and Figure 6B). UWFAF showed bilateral placoid lesions with hyperautofluorescence most prominent in the temporal macula extending into the temporal periphery in the right eye (Figure 5C) and numerous, eccentric small dots and spots in the left eye (Figure 6C). OCT showed ellipsoid zone and RPE/Bruch membrane disruption from an elevated, outer retinal hyperreflective lesion in the fovea of the right eye (Figure 5D) and a spared left macula (Figure 6D).

The patient underwent an extensive serological workup, which revealed positive syphilis antibodies. The patient was diagnosed with syphilis. One month after IV penicillin (24 million units/per day for 14 days) treatment, the patient’s visual symptoms and clinical findings resolved.

## 4. Discussion

This study supports the expanded use of UWFAF for managing syphilitic chorioretinitis and describing it within the white dot syndrome spectrum. To our knowledge, this investigation is the first case series or cohort to identify a potentially more specific finding than the classic placoid lesion—white dots and spots—in diagnosing syphilitic chorioretinitis. Building upon two isolated case reports that depict syphilitic chorioretinitis as an atypical white dot syndrome, this case series proposes the characterization of syphilitic chorioretinitis as a white dot syndrome rather than an atypical manifestation [29,30]. Torjani et al. described a case of acute syphilitic outer retinopathy (SOR) in a 51-year-old man presenting with creamy white dots extending to the retinal midperiphery, initially mimicking a white dot syndrome. Outer retinal involvement was shown through FAF imaging with hyperautofluorescent lesions corresponding to these dots. FAF was essential in distinguishing SOR from other white dot syndromes and guiding successful therapy, evident in the near-complete resolution of lesions and restoration of 20/20 visual acuity following prompt treatment with intravenous penicillin [29]. Similarly, in Case 2 of our study, the confluence and placoid appearance in the macula is suggestive of syphilitic outer retinopathy; however, other dots and spots adjacent to this lesion may support the spectrum of disease typically found with white dot syndromes. In a case of acute syphilitic posterior placoid chorioretinitis (ASPPC) reported by Hussnain et al., a 49-year-old woman exhibited hyperautofluorescent dots that were only visible on FAF imaging, demonstrating the value of multimodal imaging in identifying atypical features. FAF is useful in distinguishing syphilitic chorioretinitis from other posterior uveitic conditions, as evidenced by the patient’s considerable improvement in visual acuity and imaging abnormalities following therapy [30].

Combined with these isolated case reports, the shared features of hyperautofluorescent dots and spots observed in syphilitic chorioretinitis are distinct, consistent findings that support its inclusion within the white dot syndrome spectrum. This characterization emphasizes the importance of a low diagnostic threshold for syphilis when encountering white dots and spots, rather than exclusive reliance on placoid lesions, with multimodal imaging.

Sole dependence on IVFA for ancillary imaging could be problematic for missing syphilitic chorioretinitis. Though evidence of leakage at the optic nerve head and vasculitis may best be seen with IVFA, the recent introduction and wide clinical availability of UWFAF offers a noninvasive, efficient, and effective method in assessing RPE and neurosensory retina involvement, at a time when this infection has become increasingly prevalent [4]. Moreover, the lesions qualitatively appeared more prominent in each of these cases with UWFAF than with ultra-widefield IVFA.

While our case series shares similarities with the findings of Torjani et al. and Hussain et al., our study builds upon their work. Hussain et al. showed how FAF can be used to identify hyperautofluorescent lesions and white dots associated with syphilitic chorioretinitis but used limited standard views and only captured macular and peripapillary regions. Our case series and the case report by Torjani et al. used ultra-widefield fundus imaging and UWFAF to detect and characterize both placoid lesions and scattered white dots/spots across the retinal periphery [29,30]. The expanded view provided a more detailed assessment of the disease by identifying subtle, peripheral retinal changes, which are often missed by imaging techniques with a narrower field. By demonstrating how ultra-widefield imaging can visualize these features, our case series represents the advancements in diagnosing and monitoring syphilitic chorioretinitis, further arguing for its classification as a white dot syndrome.

Placoid appearance is traditionally a characteristic finding in syphilitic chorioretinitis. However, placoid lesions were only found in three eyes within this study, making the diagnosis more challenging with lower sensitivity. While the absence of placoid lesions does not exclude syphilis, all five eyes exhibited white dots and spots, highlighting that their presence with UWFAF imaging should prompt consideration of testing for syphilis. UWFAF provides a noninvasive, efficient, and effective means of assessing retinal involvement. By revealing hyperautofluorescence in affected areas, UWFAF facilitates earlier and more accurate diagnoses while also monitoring treatment response in real time [31,32].

Various white dot syndromes manifest with multiple dots and spots on UWFAF [33]. Multiple Evanescent White Dot Syndrome (MEWDS) often presents with small hyperautofluorescent dots in the posterior pole and ellipsoid zone disruption on OCT, both of which resemble findings in syphilitic chorioretinitis. The spontaneous resolution of these features in MEWDS parallels the post-treatment improvements seen in syphilitic chorioretinitis [34]. Acute Posterior Multifocal Placoid Pigment Epitheliopathy (APMPPE) is characterized by placoid lesions on FAF and IVFA. This condition shares imaging similarities with syphilitic chorioretinitis, particularly in the acute phase [35]. Punctate Inner Choroidopathy (PIC) shows hyperautofluorescent dots on FAF and outer retinal disruption on OCT, similar to syphilitic chorioretinitis, though without vitritis [36].

When comparing syphilitic chorioretinitis to these findings, the hyperautofluorescent dots and spots observed on UWFAF closely resemble the hallmark findings in MEWDS, APMPPE, and PIC. These dots and spots likely indicate retinal pigment epithelial disruption and align with imaging features seen across white dot syndromes [22,23,26]. OCT findings in syphilitic chorioretinitis, like the disruption of the ellipsoid zone and RPE with or without outer retinal thinning, are consistent with changes observed in MEWDS and Acute Zonal Occult Outer Retinopathy (AZOOR) [37,38,39]. These shared findings portray a diagnostic overlap.

Despite their presence in many white dot syndromes, the lesions identified in all three cases most closely overlap with MEWDS and are predominantly in the posterior pole. At presentation, both disease processes demonstrate white spots most evident on UWFAF and corresponding outer retinal changes visible on OCT imaging. Case 2 portrays the resolution of the white dots/spots and outer retinal changes seen on OCT after treating syphilis, similar to spontaneous improvement in MEWDS [34]. The presence or persistence of white dots and spots on UWFAF may prompt further evaluation for syphilitic chorioretinitis [13,32,40]. Categorizing syphilitic chorioretinitis as a white dot syndrome could highlight its diagnostic consideration when it may otherwise be undetected.

Although the exact pathologic mechanism of scattered pinpoint hyperautofluorescent regions is unknown, we suspect the white dots represent either inflammation or spirochete infiltrates within the outer retina and RPE. Dots and spots observed in syphilitic chorioretinitis may be attributed to the direct invasion of spirochetes into the lesions. While studies have hypothesized the presence of *Treponema pallidum* within chorioretinal lesions, direct histopathological confirmation is lacking [33]. Conversely, immune status also affects the appearance of white dots and spots, indicating that a secondary immune response may be present [11]. The role of inflammatory processes in the context of ocular syphilis was supported by Balaskas et al.’s study, which showed that dark dots observed during fundus indocyanine green angiography (ICGA) were significantly associated with anterior uveitis [31]. Similarly, Knecht et al. reported ICGA patterns consistent with choriocapillaritis in syphilitic chorioretinitis [41]. Further supporting the inflammatory hypothesis, Eandi et al. also reported the resolution of these lesions following antibiotic therapy [42]. This inference aligns with findings in other white dot syndrome conditions, where immune-mediated processes are thought to drive disease manifestations [28].

Other studies have proposed this observed phenomenon is associated with outer retinal disruption rather than alteration within the RPE [24], or increased autofluorescence linked to outer retinal disruption may be due to a window defect caused by photopigment loss [43]. These mechanisms might occur independently or concurrently, depending on the extent of spirochete invasion and the host immune response. Mirzania et al. demonstrated that RPE foci, like placoid lesions, demonstrate reversibility post-treatment and can lead to photoreceptor dysfunction and decreased visual acuity [44].

Syphilitic chorioretinitis is thought to have an overlapping inflammatory and infectious process, much like other retinal diseases. An immune-mediated choroidal inflammation, for example, has been associated with white dot syndromes like MEWDS, indicating a potential shared pathway [28]. We suspect, as have others, that the inflammation of the outer retinal structures from direct spirochete may overlap with autoimmune conditions, which would explain a comparable phenotype in these cases. The need for further research is highlighted by the complex pathophysiology of syphilitic chorioretinitis.

Although this study presents strong evidence to support the characterization of syphilitic chorioretinitis as a white dot syndrome, it is important to address potential limitations and alternative viewpoints. First, white dot syndromes are traditionally noninfectious and associated with autoimmune or viral triggers. Despite different etiologies, syphilitic chorioretinitis shares significant clinical and imaging overlap with white dot syndromes. For example, both frequently exhibit visual symptoms such as photopsias and floaters, ellipsoid zone disruption on OCT, and hyperautofluorescent dots on UWFAF [22,38,39]. Secondly, syphilitic chorioretinitis requires systemic antibiotic therapy, in contrast to white dot syndromes that are typically self-limited or require immunosuppression. However, the white dot syndrome spectrum already includes conditions with varying prognoses and treatment approaches. MEWDS often resolves spontaneously, while multifocal choroiditis and panuveitis (MCP) requires aggressive immunosuppression to prevent complications such as choroidal neovascularization [45]. Additionally, testing for HIV in patients with syphilitic chorioretinitis is important due to the prevalence of co-infection, with routine testing recommended and, in some jurisdictions, mandated; however, HIV testing cannot be confirmed for the patients in this study. Lowering the diagnostic threshold for syphilitic chorioretinitis can be achieved with this characterization by prompting clinicians to test for syphilis in patients with white dot-like findings. Multimodal imaging can help achieve early diagnosis, which is essential to avoiding irreversible visual and systemic complications [6,23].

Characterizing syphilitic chorioretinitis as a white dot syndrome promotes a broader understanding of retinal inflammatory processes and challenges the boundaries of the white dot syndrome spectrum. While urging clinicians to use advanced imaging modalities to improve patient outcomes, this characterization also encourages clinicians to consider infectious etiologies in cases of white dot/spot-like presentations.

## 5. Conclusions

While it is challenging to diagnose “the great masquerader” due to its diverse range of symptoms and presentations, this study demonstrates the diagnostic utility of ultra-widefield imaging in syphilitic chorioretinitis and highlights its considerable overlap with white dot syndromes. In this study, we show that ultra-widefield fundus autofluorescence (UWFAF) adds to conventional and multimodal imaging capabilities in diagnosing syphilitic chorioretinitis through hyperautoflorescence from the affected retina and retinal pigment epithelium. The distinct dots/spots found with UWFAF as part of a multimodal approach in syphilitic chorioretinitis may occur more frequently than classic placoid lesions, which align with imaging features of conditions like MEWDS, APMPPE, and PIC, and therefore support its characterization as a white dot syndrome. Even in cases lacking placoid lesions, the presence of white dots and spots supports their use as diagnostic biomarkers. The diagnostic threshold for syphilis will be lowered in cases of unexplained posterior uveitis or white dot-like findings if syphilitic chorioretinitis is characterized within the white dot syndrome spectrum. Due to the rising prevalence of *Treponema pallidum*, the characterization of syphilitic chorioretinitis as a member of this spectrum highlights the importance of early laboratory diagnosis and timely antibiotic treatment. To ensure prompt diagnosis and treatment, it is essential to raise awareness of this disorder among ophthalmologists as well as family medicine physicians and infectious disease specialists.

Future research should focus on validating these findings in larger cohorts and exploring the underlying pathophysiological mechanisms of hyperautofluorescent lesions in syphilitic chorioretinitis. This study provides a foundation for more effective diagnosis and management of syphilitic chorioretinitis.

## Data Availability

The original contributions presented in this study are included in the article. Further inquiries can be directed to the corresponding author.

## Abbreviations

The following abbreviations are used in this manuscript:

UWFAF	directory of open access journals
OCT	optical coherence tomography
IVFA	intravenous fluorescein angiography
CFP	ultra-widefield pseudocolor fundus photography
MEWDS	multiple wvanescent white dot syndrome
ARPE	acute retinal pigment epitheliitis
APMPPE	acute posterior multifocal placoid pigment epitheliopathy
MCP	multifocal choroiditis and panuveitis
AZOOR	acute sonal occult outer retinopathy
PIC	punctate inner choroidopathy
